# Prevalence of hepatotoxicity among HIV-infected patients in Ethiopia: a systematic review and meta-analysis

**DOI:** 10.1186/s12879-022-07838-w

**Published:** 2022-11-09

**Authors:** Ousman Mohammed, Ermiyas Alemayehu, Habtye Bisetegn, Mihret Tilahun, Alemu Gedefie, Endris Ebrahim, Mesfin Fiseha, Mogesie Necho, Temesgen Fiseha

**Affiliations:** 1 Department of Medical Laboratory Sciences, College of Medicine and Health Sciences, Wollo University, Dessie, Ethiopia; 2 Department of Psychiatry, College of Medicine and Health Sciences, Wollo University, Dessie, Ethiopia

**Keywords:** Hepatotoxicity, Liver enzyme elevation, Antiretroviral therapy, HIV/AIDS, Ethiopia

## Abstract

**Background:**

Globally, the human immunodeficiency virus has been recognized as a major public health concern. The direct toxicity of antiretroviral medicines or their active metabolites causes liver cell destruction by different mechanisms, inducing immune-mediated inflammation, oxidative stress, and other mechanisms. On the other hand, the virus itself also produces hepatotoxicity. Therefore, this systematic review and meta-analysis aimed to assess the pooled prevalence of hepatotoxicity among HIV-infected patients in Ethiopia.

**Methods:**

PubMed, Science Direct, Cochrane Library, Web of Science, and ResearchGate databases were used to find relevant articles. As well, various professional associations were searched to retrieve grey literature. The Newcastle–Ottawa Quality Assessment Scale was used to assess the quality of recruited studies. The data were extracted using Microsoft Excel, and the meta-analysis was carried out using STATA 14 software. I^2^ and Cochran’s Q test were employed to assess the presence of heterogeneity between studies. A random effect model was used. The funnel plot and Egger’s statistics were used to assess publication bias. Moreover, subgroup analysis and sensitivity analysis were also done.

**Results:**

The pooled prevalence of hepatotoxicity among HIV patients in Ethiopia was 25.45% (95% CI = 20.06–30.84%). There was high heterogeneity, with an I^2^ value of 93.7%. Subgroup analysis by HAART status showed a higher pooled prevalence of hepatotoxicity among HIV patients taking HAART (23.63%) than among HAART naive patients (7.29%). In subgroup analysis, the pooled prevalence of hepatotoxicity among HIV/Tb co-infected and HIV mono-infected patients was 26.3% and 17.94%, respectively.

**Conclusion:**

The current systematic review and meta-analysis showed a high prevalence of hepatotoxicity among HIV-infected patients. Therefore, regular monitoring of hepatotoxicity among HIV-infected patients is required in order to avoid liver damage and other complications.

*Systematic review registration* PROSPERO (2022:CRD42022334704)

**Supplementary Information:**

The online version contains supplementary material available at 10.1186/s12879-022-07838-w.

## Introduction

Since the beginning of the twenty-first century, the human immunodeficiency virus (HIV) has been recognized as a major public health concern around the world [1, 2]. According to figures from 2019, HIV/AIDS is a pandemic disease with 38.0 million people living with the virus worldwide. Sub-Saharan Africa accounts for around 25.7 million of these people. In addition, 730,000 new HIV infections were reported in Eastern and Southern Africa in 2019 [3]. To meet the UNAIDS 90-90-90 targets, most African countries will have integrated community-based interventions into HIV healthcare service delivery by the beginning of 2020. Significant progress has been made in Sub-Saharan Africa in meeting the UNAIDS 90-90-90 objectives by the end of 2019. However, several African countries, like Ethiopia, have not yet met these targets [4]. By 2019, 87% of people living with HIV (PLHIV) in Eastern and Southern Africa recognized their status, 72% of PLWH were on treatment, and 65% of PLHIV in Eastern and Southern Africa were virally suppressed [5].

In HIV-infected patients, non-AIDS causes of morbidity and mortality are becoming more common. Liver illnesses have risen to become one of the top causes of death not related to AIDS. When evaluating and caring for these patients, a full understanding of the mechanisms underlying the development of liver disease is critical. HIV-related morbidity and mortality have decreased, and the burden of opportunistic infections (OIs) has declined significantly since the advent of highly active antiretroviral treatment (HAART) [6]. Because of the rapid scale-up of antiretroviral therapy, HIV-related morbidity and mortality have decreased considerably, even in underdeveloped countries [7].

Despite the fact that Ethiopia has had a free ART service since 2005, the prevalence of HIV and other HIV-related diseases such as hepatotoxicity is still too high [8]. HAART enhanced the quality of life of HIV patients by considerably reducing the frequency of opportunistic infections, morbidity, and mortality, despite a considerable change in the quality of life [9, 10]. However, drug-induced liver disease, hyperlipidemia, hyperglycemia, and lactic acidosis are still typical HAART adverse effects [11]. Because of asymptomatic or symptomatic side effects, patients commonly discontinue medication [12–14]. Poor adherence to HAART increases the chance of drug-resistant virus strains developing and can result in hepatotoxicity [15, 16].

Measuring liver enzymes is the most common way of determining hepatotoxicity. Hepatotoxicity grades are broadly classified as mild when an ALT value is between 1.25 and 2.5 upper reference limit, moderate when it is between 2.5 and 5.0 upper reference limit, and severe when it is between 5.0 and 10 upper reference limit [17, 18]. HIV and antiretroviral medications both cause abnormalities in liver enzymes. Biochemical monitoring revealed aberrant liver functions and symptoms in patients, indicating that anti-HIV medications should be stopped, and anti-HIV treatments were temporarily stopped due to hepatotoxicity [19].

Antiretroviral (ARV) medications harm liver cells either directly or through their active metabolites. On the other hand, immune-mediated injury, oxidative stress, mitochondrial injury, lipotoxicity, cytotoxicity, toxic metabolite build-up, gut microbial translocation, and systemic inflammation, on the other hand, are among HIV viral pathogenesis and liver injury mechanisms [12]. Because of the strong link between HIV and tuberculosis, all antiretroviral medicines, as well as several anti-TB drugs, have prevalent and problematic side events that cause adherence issues, hospitalization, and the risk of hepatotoxicity [20–22].

Toxicity may be linked to the drug's dose in cases of predicted liver injury. Patients on nevirapine-containing regimens, for example, experienced liver damage and skin rashes [23]. Studies indicate that several ARV medications have been linked to hepatotoxicity and nephrotoxicity [24, 25]. Despite the fact that numerous studies have been undertaken in various parts of Ethiopia, there is a lack of consistency in the extent and overall incidence of HAART and HIV virus-related hepatotoxicity. There has never been a study that establishes the overall prevalence of hepatotoxicity in HIV-positive patients. As a result, this was the first of its kind to be a systematic review and meta-analysis with the purpose of presenting comprehensive data on the prevalence of hepatotoxicity.

## Methods

### Protocol registration

The current systematic review and meta-analysis were conducted in accordance with the Preferred Reporting Items for Systematic Review and Meta-Analyses (PRISMA) statement (Additional file 1: Table S1) [26]. The study protocol was registered at the International Prospective Register of Systematic Reviews (PROSPERO) with the registration number (2022:CRD42022334704).

### Search strategy

A standard search strategy is used in PubMed, and later it is modified according to each specific database to get the best relevant results. Search strategies are constructed to include free-text terms (e.g., in the title and abstract) and any appropriate subject indexing (e.g., MeSH) expected to retrieve eligible studies, with the help of an expert in the review topic field. The three reviewers systematically searched different databases like Google Scholar, PubMed, Science Direct, Cochrane Library, EMBASE, Web of Science, and ResearchGate from March to May 22, 2022, using the combination of following keywords: “hepatotoxicity’’ OR “liver enzyme elevation” OR “biochemical alteration” OR “antiretroviral therapy” AND “HIV/AIDS” AND Ethiopia. Furthermore, grey literature was systematically searched in professional associations such as the Ethiopian Public Health Association (EPHA) and the Ethiopian Medical Laboratory Association (EMLA), university databases, and national annual conferences. The references from the retrieved articles, relevant reviews, and previous meta-analyses were searched for additional studies not identified by the database search. Searched articles were entered into Endnote Software to avoid duplicates and the list was consolidated into one list. The remaining articles were screened for their titles and abstracts independently by reviewers. Any disagreement during screening was resolved by consensus (Additional file 3).

### Inclusion and exclusion criteria

The inclusion and exclusion criteria were determined after formulating the research question by using PI/ECO (Population, Intervention, Exposure, Comparison, and Outcome). Only original studies that reported the prevalence of hepatotoxicity among HIV/AIDS patients in Ethiopia were included in this systematic review and meta-analysis. In addition, studies reporting the prevalence of hepatotoxicity among HIV/AIDS patients co-infected with *Mycobacterium tuberculosis (TB)* were also included. These are articles with information that answers our research question. Likewise, no restrictions were applied regarding region, patient age, gender, and date of publication. We have narrowed our search to literature written in English. For duplicate studies, the first version or the one with all the necessary data was used. Exclusion criteria are mostly unrelated, duplicated, unavailable full texts, abstract-only papers, case reports, case series, letters to the editor, conference proceedings, or review pieces.

### Data extraction and quality assessment

Three reviewers (Ousman M., Ermiyas A., and Habtye B.) independently selected the studies using the eligibility criteria based on the title, abstract, and full text. These reviewers then retrieved data from the entire text of potentially eligible studies using standardized data extraction forms compared the results, and resolved inconsistencies through consensus-based discussions. For identification purposes, each study was assigned a unique number, and the following descriptive data was extracted: name of the principal author; year of publication; regions where the study was conducted; methods (design and settings), total sample size; overall prevalence of hepatotoxicity; prevalence of hepatotoxicity before HAART; prevalence of hepatotoxicity after HAART; HIV/Tb co-infection status; and grading of hepatotoxicity (mild to severe). When studies lacked enough methodological information or the information was confusing, the authors were approached via an official email address or phone number for clarification. Three independent reviewers assessed the quality of the included studies using the Newcastle–Ottawa Quality Assessment Scale for recruited studies (NOS) [27]. Cohort studies’ quality was assessed using a 9-point scoring system, while cross-sectional and case–control studies' quality was assessed using an 8-point scoring system (Additional file 2: Table S2-S4).

### Statistical analysis

The final meta-analysis was carried out using STATA version 14 using the metan commands. Because of the high heterogeneity, the pooled effect size and 95% confidence interval (95% CI) were calculated using a random-effects meta-analysis model. The I^2^ statistic and Cochran’s Q test with its related p-value were used to analyse the heterogeneity among studies. For a pooled analysis, an I^2^ statistic value of less than 25% was equivalent to no heterogeneity, 25–50% was equivalent to low heterogeneity, more than 50% was considered moderate, and 75% was considered high heterogeneity [28, 29]. The aggregate prevalence and 95% CI were presented using forest plots. Subgroup analysis was performed according to the publication year, study design, HIV/Tb co-infection status, ART status, and hepatotoxicity grading to investigate the source of the substantial heterogeneity reported. We ran a sensitivity analysis to see how a single study affected the pooled effect size. Any asymmetry in a funnel plot, as well as statistical significance in Egger’s regression test (p-value < 0.05), indicated publishing bias. As a result, the random effect analysis was used to do a nonparametric trim and fill analysis [30].

## Results

### Description of included studies

**Fig. 1:**
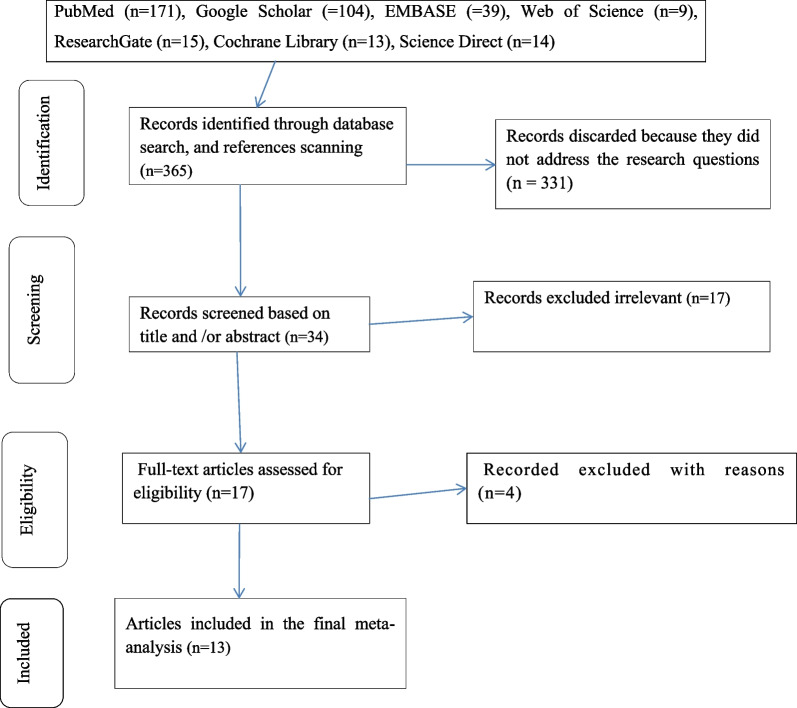
Flow chart of studies’ search and retrieval process

The current systematic review and meta-analysis found 365 abstracts and citations using electronic and reference scanning searches. Three hundred and fifty-two (352 total) studies were removed after duplicates and irrelevant studies were also removed based on titles and abstracts. The articles that were included were of good quality. Finally, a total of 13 studies were included in this systematic review and meta-analysis. The steps in the article's search and retrieval procedure are depicted in (Fig. 1).

### Characteristics of the included studies

In this study, 13 original articles consisting of 3676 study participants were included [31–43]. The research was carried out in Ethiopia’s three (3) regions and one city administration. The regional breakdown of the studies was as follows: Five investigations were conducted in the Amhara area; one in Tigray; one in the South region; and the remaining six in Addis Ababa, Ethiopia’s capital. The earliest study [43] was published in 2008, and the most current study [36] was published in 2021. The sample size of the included studies varied from 84 to 1060 participants. Eight studies reported the prevalence of hepatotoxicity among patients with both HIV and TB infections, while the other five studies reported the prevalence of hepatotoxicity among HIV patients. The study participants’ mean or median age ranged from 32 to 39.7 years (Table 1).

**Table 1:** The characteristics of the studies those were included in Ethiopia, 2022

Authors (Year)	Region	Study design	Mean/median age (year)	Sample size	HIV/Tb co-infection status	Total prevalence of hepatotoxicity (%)	Overall hepatotoxicity on treatment naïve (%)	Overall hepatotoxicity on ART (%)	Degree of hepatotoxicity		
									Mild (%)	Moderate (%)	Severe (%)
Yazie (2021) [31]	Addis Ababa	Prospective cohort	39.7 ± 10	63	HIV	41.3	NA	41.3	38.2	3.2	NA
Baynes et al. (2017) [32]	Amhara	Retrospective cohort	NA	275	HIV	23.3	6.5	23.2	2.2	10.9	3.6
Shiferaw et al. (2016) [33]	Amhara	Cross-sectional	36.29 ± 10.27	328	HIV	12.2	22	20.1	6.7	3.7	3
Mulu et al. (2013) [34]	Amhara	Cross-sectional	35	269	HIV	32	NA	32	22.3	7.8	1.84
Tesfa et al. (2019) [35]	Amhara	Cross-sectional	37.37	152	HIV	17.1	9.2	25	23.7	1.3	NA
Gebremicael et al. (2021) [36]	Addis Ababa	Prospective cohort	32	316	HIV/Tb	29.1	22.8	38	21.7	9.6	1.2
Woldu et al. (2014) [37]	Tigray	Case–control	33.6 ± 17	100	HIV/Tb	60	NA	55	45	8	7
Yimer et al. (2014) [38]	Addis Ababa	Prospective cohort	NA	1060	HIV/Tb	15	NA	15	8.02	4.9	2.08
Yimer et al. (2011) [39]	Addis Ababa	Prospective cohort	NA	353	HIV/Tb	30	NA	30	NA	11.6	18.4
Yimer et al. (2012) [40]	Addis Ababa	Prospective cohort	34	285	HIV/Tb	24.1	8.4	15.7	NA	11.6	18.4
Hassen Ali et al. (2013) [41]	Oromia	Case–control	32.1 ± 8.5	288	HIV/Tb	11.5	NA	NA	6.3	NA	5.2
Zeleke et al. (2020) [42]	Amhara	Cross-sectional	NA	84	HIV/Tb	20.2	NA	NA	NA	NA	NA
Yimer et al. (2008) [43]	Addis Ababa	Prospective cohort	NA	103	HIV/Tb	25.2	NA	NA	NA	NA	NA

*NA* Not available

### Prevalence of hepatotoxicity among HIV infected patients

**Fig. 2:**
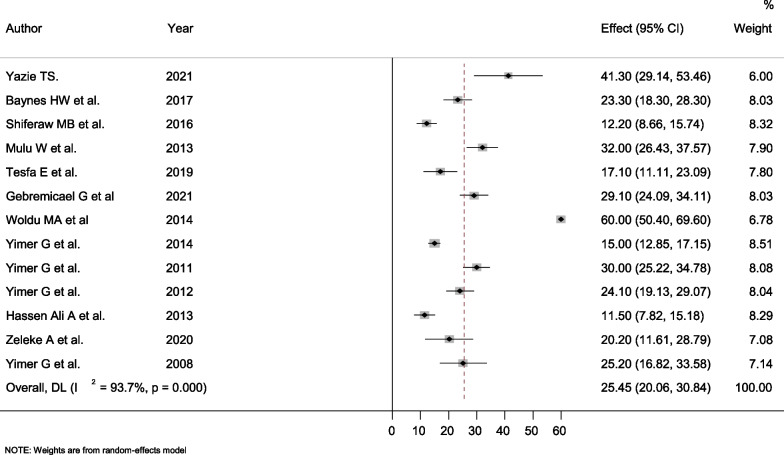
Forest plot showing the pooled prevalence of hepatotoxicity among HIV infected patients

In this study, the prevalence of hepatotoxicity ranged from 11.5% reported in Jimma [41] to 60% reported in Mekele [37]. The current systematic review and meta-analysis indicated that the overall pooled prevalence of hepatotoxicity among HIV-infected patients in Ethiopia was 25.45% (95% CI = 20.06–30.84%). High heterogeneity was observed with a value of Q test (Tau-squared) of 87.73 (degree of freedom, d.f = 12, p-value < 0.001), and I^2^ was determined as 93.75% for the degree of inconsistency (Fig. 2).

### Subgroup analysis

#### ART status

**Fig. 3:**
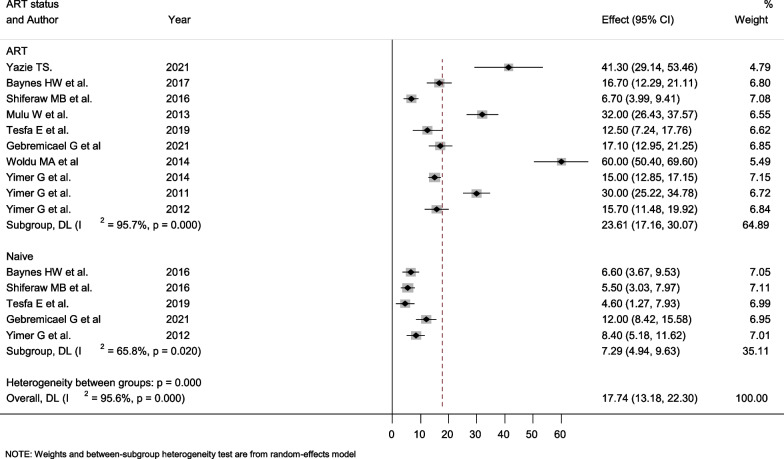
Forest plot showing the pooled prevalence of hepatotoxicity among ART taking and naïve HIV/AIDS patients

To investigate the source of high heterogeneity, subgroup analysis was done based on ART status, hepatotoxicity grade, study design, publication year, and HIV/TB co-infection. The prevalence of hepatotoxicity among treatment naive and treatment-taking groups has not been stated in all the investigations. The prevalence of hepatotoxicity among treatment-naive patients ranged from 4.6% (95% CI; 1.27–79.93%) to 8.4% (95% CI; 5.18–11.62%) with a pooled prevalence of 7.29% (95% CI; 4.94–9.63%). On the other hand, the pooled prevalence of hepatotoxicity among treatment-taking patients was exceptionally high. The computed prevalence ranges from 6.7% (95% CI; 3.99–9.41%) to 60% (95% CI; 50.4–69.6%) with a pooled prevalence of 23.61% (95% CI; 17.16–30.07%). Treatment-taking and treatment-naive groups’ also demonstrated substantial heterogeneity, with a value of I^2^ of 95.7% and 95.6%, respectively (Fig. 3).

### Subgroup analysis by degree of hepatotoxicity

**Fig. 4:**
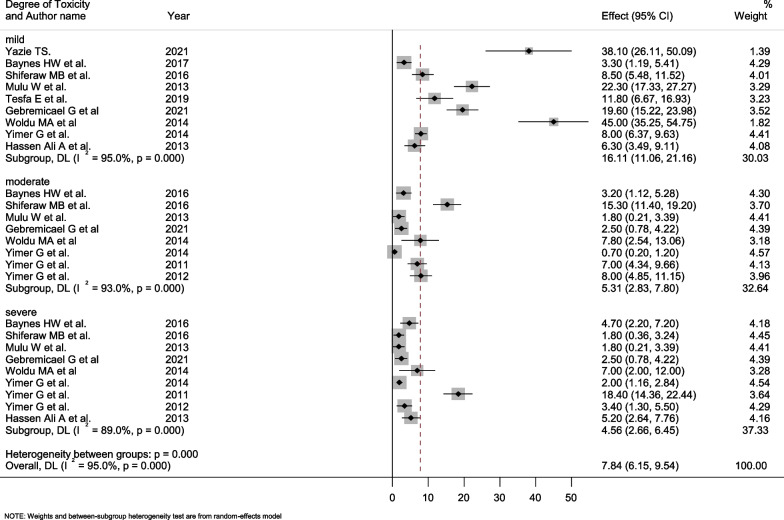
Forest plot showing the pooled prevalence of hepatotoxicity grading among treatment taking and naïve HIV/AIDS patients

In this meta-analysis, the selected studies had the highest prevalence of mild hepatotoxicity, ranging from 3.3% (95% CI: 1.19–5.41) to 45.0% (95% CI: 35.25–54.75), with a pooled prevalence of 16.11% (95% CI: 11.06–21.16). The pooled prevalence of severe hepatotoxicity among the reported studies was 4.56% (95% CI: 2.66–6.45), which varied from 1.8% (95% CI: 0.21–3.39) to 18.4% (95% CI: 14.36–22.44). All levels of hepatotoxicity showed significant heterogeneity (Fig. 4).

### Subgroup analysis by study design and year of publication

**Fig. 5:**
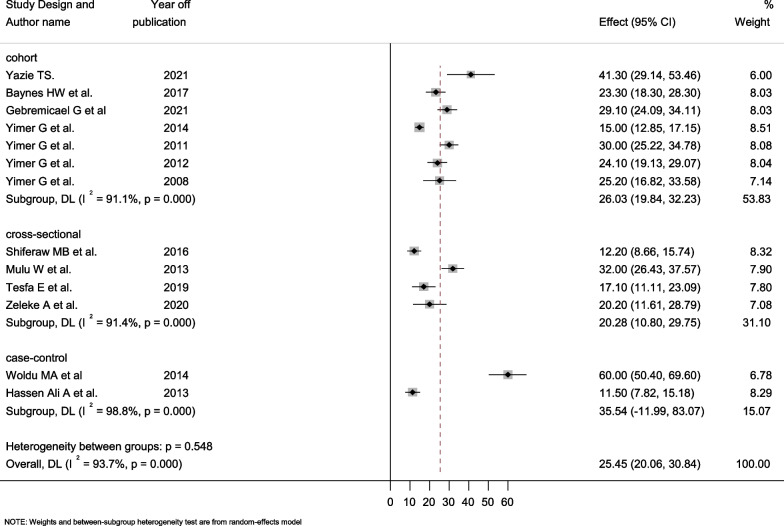
Forest plot showing the pooled prevalence of hepatotoxicity among HIV/AIDS patients by study design

**Fig. 6:**
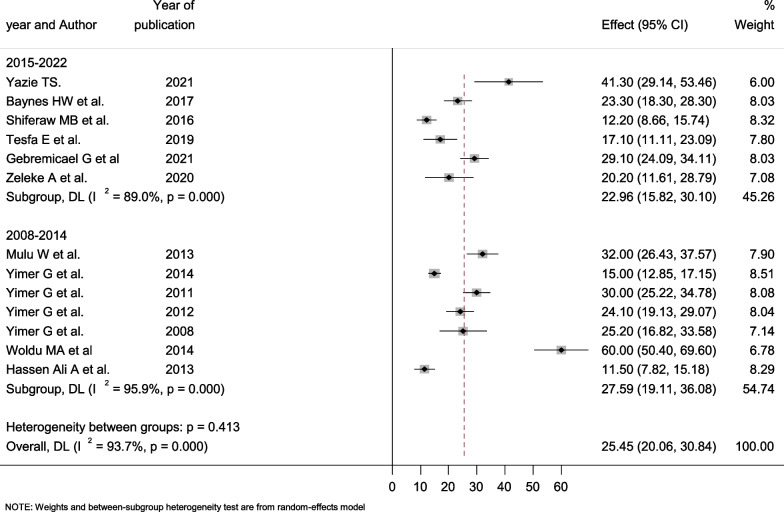
Forest plot showing the pooled prevalence of hepatotoxicity among HIV/AIDS patients by year of publication

Seven studies employed cohort study designs, four were cross-sectional, and the remaining two used case–control study designs. The case–control studies' estimated prevalence of hepatotoxicity ranged from 11.5% (95% CI: 7.82–15.18) to 60.0% (95% CI: 50.40–69.60) with a pooled prevalence of 35.54% (95% CI: 11.99–83.07). The determined pooled prevalence was lower among cross-sectional studies at 20.28% (95% CI: 10.80–29.75), which varied from 12.2% (95% CI: 8.66–15.74) to 32% (95% CI: 26.45–37.57). In all designs, there was markedly high heterogeneity, with an I^2^ value of 91.1% and 98.8% Fig. 5. Moreover, we grouped all the studies into two groups based on their publication year, i.e., 2008–2014 and 2015–2022. The pooled prevalence of hepatotoxicity was 27.59% (95% CI: 19.11–36.08) and 22.29% (95% CI: 15.82–30.10) in studies conducted between 2000 and 2014. According to the results from this meta-analysis, the pooled prevalence of hepatotoxicity decreased somewhat from 2008 to 2014 to 2015–2022. There was significant variation within and across groups (Fig. 6).

### Subgroup analysis by HIV/Tb Co-infection status

**Fig. 7:**
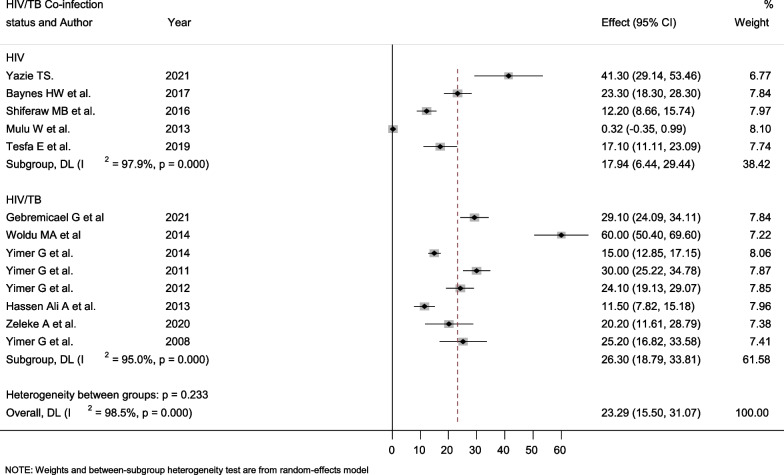
Forest plot showing the pooled prevalence of hepatotoxicity among HIV/AIDS patients by HIV/Tb Co-infection status

The majority of the studies included in this meta-analysis reported hepatotoxicity among HIV/TB co-infected patients. The current meta-analysis found that HIV/TB co-infected patients had a higher pooled prevalence of hepatotoxicity than HIV mono-infected patients [26.3% (95% CI: 18.79–33.81)] versus [17.94% (95% CI: 6.44–29.44)]. In both groups, high levels of heterogeneity I^2^ were observed (Fig. 7).

### Publication bias

**Fig. 8:**
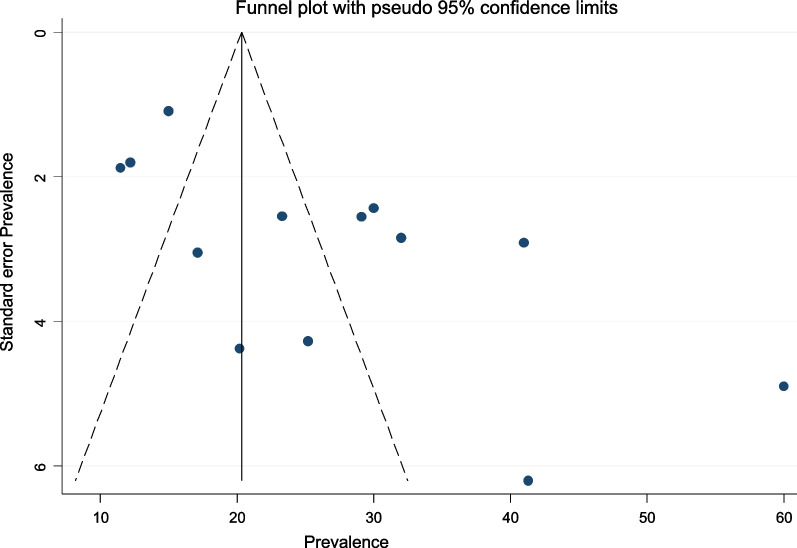
Bias assessment plot of reported prevalence rates of hepatotoxicity among HIV-infected individuals across studies published in Ethiopia between 2008 and May, 2022

The prevalence of hepatotoxicity among HIV patients in this meta-analysis did not show a totally symmetrical depiction of the prevalence reported by the various studies. Egger's test statistics also indicated the presence of publication bias with a p-value of 0.006. This was also supported by the asymmetry of the funnel plot (Fig. 8).

### Trim and fill analysis of pooled prevalence of hepatotoxicity among HIV-infected patients

Based on trim and fill analysis, after adding six studies, the pooled prevalence of hepatotoxicity among HIV-infected in Ethiopia was 16.42% (95% CI 10.63–22.20) at a p-value < 0.001 (Table 2).

**Table 2:** Trim and fill analysis of overall pooled prevalence of hepatotoxicity among HIV-infected patients in Ethiopia, 2022

Meta-analysis^a^					
Method	Pooled est	95% CI	Z-value	p-value	No. of studies
Fixed	19.58	18.33–20.83	30.683	< 0.001	13
Random	25.45	20.06–30.84	9.250	< 0.001	

Iteration	Estimate	Tn	# to trim	Diff
1	19.58	69	4	91
2	16.94	80	6	22
3	15.71	83	6	6
4	15.71	83	6	0

Filled meta-analysis^b^					
Method	Pooled est	95% CI	Z-value	p-value	No. of studies
Fixed	15.71	14.58–16.84	27.32	< 0.001	19
Random	16.416	10.63–22.20	5.558	< 0.001	

^a^Test for heterogeneity: Q = 191.844 on 12 degrees of freedom (p = 0.000)

Trimming estimator: Linear

Meta-analysis type: Fixed-effects mode

^b^Test for heterogeneity: Q = 426.787 on 18 degrees of freedom (p ≤ 0.001)

Moment-based estimate of between studies variance = 153.14

### Sensitivity analysis

A sensitivity analysis was used to see how a single study affected the pooled effect size. When one study was removed at a time, the resulting pooled effect size was within the 95% confidence interval of the combined pooled effect size, indicating that no single study influenced the impact size (Table 3).

**Table 3:** Sensitivity analysis

S. No.	Study omitted	Estimate	95% CI
1	Yazie (2021)	24.41	18.98–29.85
2	Baynes et al. (2017)	25.69	19.86–31.52
3	Shiferaw et al. (2016)	26.69	20.89–32.49
4	Mulu et al. (2013)	24.86	19.34–30.37
5	Tesfa et al. (2019)	26.20	20.42–31.97
6	Gebremicael et al. (2021)	25.14	19.50–30.78
7	Woldu et al. (2014)	22.75	18.18–27.31
8	Yimer et al. (2014)	26.54	20.36–32.72
9	Yimer et al. (2011)	25.04	19.45–30.63
10	Yimer et al. (2012)	25.62	19.80–31.43
11	Hassen Ali et al. (2013)	26.74	21.00–32.47
12	Zeleke et al. (2020)	25.87	20.19–31.56
13	Yimer et al. (2008)	25.49	19.82–31.15
14	Combined	25.45	20.06–30.84

## Discussion

Antiretroviral therapy (ART) has considerably reduced morbidity and death among HIV-positive people around the world [44]. By 2016, an estimated 19.5 million people had gotten ART around the world [45]. ART treatment, despite its enormous health benefits, has some side effects, particularly hepatic impairment, which contributes to morbidity and mortality in HIV patients [46, 47]. In HIV-infected patients, multiple mechanisms of liver damage have been identified, including HIV infection [48], hepatitis virus co-infections, ART-related neurotoxic effects [49], AIDS-related neoplasm [50], and age-related co-morbid conditions [51].

There is no published study that assesses the nationwide pooled prevalence of hepatotoxicity among HIV-infected individuals that we are aware of. As a result, this meta-analysis was the first to present comprehensive information on the magnitude of hepatotoxicity in HIV-positive people. In almost all of the investigations, the occurrence of hepatotoxicity was not associated with age or sex. According to our findings, the countrywide prevalence of hepatotoxicity among HIV-infected patients was 25.45% (95% CI = 20.06–30.84%). However, it differs from other cohort studies in Uganda (4.2%) [52], Botswana (1.1%) [53], the Netherlands (7.9%) [54], Taiwan (4.9%) [55], a randomised trial in Boehringer Ingelheim (10%) [56], and South Africa (17%) [23], and a review done by Kontorinis N et al. found (18%) [57]. In contrast, a study in Cameroon (42.1%) [58], Nigeria (36.4%) [19], and Iran (32% [59]) found a high prevalence of hepatotoxicity. The difference could be due to the differences in the prevalence of risk factors for liver disease like hepatitis B and C and other opportunistic infections; indiscriminate use of drugs; hepatotoxicity used; follow-up duration; and the definition criteria of hepatotoxicity. The increased prevalence in the current study may be brought on by additional hepatotoxicity risk factors.

This high risk of hepatotoxicity highlights the necessity for a national policy that includes hepatotoxicity screening tests as part of comprehensive care for HIV patients. Furthermore, the extent of hepatotoxicity was found to vary significantly in these investigations, ranging from 11.5% in Oromia, Jimma [41] to 60%in Tigray, Mekele [37]. As a result, there was a lot of heterogeneity (I^2^ = 93.7%). This disparity could be explained by changes in sample size, study design, the definition of high liver enzyme levels, data processing method, and study participants’ socio-demographic and clinical features. The gap may also be due to the small number of studies that were conducted; only one study was conducted in each of Oromia and Tigray, and most of them were in Addis Ababa and the Amhara region.

The random effect pooled prevalence of hepatotoxicity was higher in study patients who were on treatment at 23.61% (95% CI; 17.16–30.07%) compared to their treatment-naive counterparts at 7.29%, according to the subgroup analysis by ART status (95% CI; 4.94–9.63%). The rate of hepatotoxicity (23.61%) in the current study was lower than that observed in patients with HIV who had received ART in Nigeria (74.2%) [60], Iran (33%) [59], and similar to that in Cameroonian patients (22.6%) [61]. The current result was higher than a study done by Raffaele et al. who reported that (2.9%) of the treatment receiving group experienced hepatotoxicity [62]. Moreover, the prevalence of hepatotoxicity among treatment-naive individuals in the current study was lower than that seen in Tanzanian patients (13%) [63] and North American patients (15%) [64].

According to these findings, the use of HAART has significantly increased hepatotoxicity in HIV-infected patients. It is undeniable that the use of HAART has drastically improved the quality of life of HIV-infected people by extending their lives. The main problems of HAART in long-term usage include the potential for hepatocyte derangements due to the drug’s direct toxicity, functional disturbance, mitochondrial injury, and/or active metabolites, all of which can be life-threatening. The virus causes hepatotoxicity by causing systemic inflammation or oxidative stress pathogenesis, and the condition is further exacerbated by HAART hepatocyte damage [65, 66]. There was also a considerably higher variability in both the medication taking and treatment naive groups, with I^2^ of 95.7% and 95.6%, respectively. A prior study found that 14–20% of HIV-infected individuals receiving antiretroviral medication (ART) experienced hepatotoxicity [67, 68].

Hepatotoxicity, which can range from mild to severe, is a major health problem that leads to high rates of morbidity, mortality, and hospitalization [69]. The current meta-analysis found that the pooled prevalence of mild hepatotoxicity was 16.11%, which was lower than the studies in Iran (23.1%) [59], and Nigeria (37.2%) [19]. Of the studies used for this review, the highest rate of mild hepatotoxicity was 45% in a study conducted by Woldu et al. in the Tigray region [37], followed by 38.1% in Addis Ababa by Yazie [31]. In the Amhara region, Baynes HW et al. found the lowest incidence of mild hepatotoxicity [32]. On the other hand, the overall rate of severe hepatotoxicity was 4.56% (95% CI: 2.66–6.45). This result was higher than the studies in Iran (1.5%) [59], the USA (2%) [70], and Nigeria (3.2%) [19]. Furthermore, previous studies found that the prevalence of hepatotoxicity ranged between 1 and 18% [71–73]. Of the studies recruited for this review, Yimer et al. found the highest rate of severe hepatotoxicity at 18.4% [39], whereas Shiferaw et al. found the lowest at 1.8% [33]. The findings underlined the difficulty in assessing and controlling hepatotoxicity associated with antiretroviral therapy and viral hepatotoxicity [74].

In the two case–control studies, subgroup analysis revealed a high pooled overall prevalence of hepatotoxicity of 35.54% (95% CI: 11.99–83.07). In contrast, cross-sectional studies reported the lowest overall frequency of hepatotoxicity at 20.28% (95% CI: 10.80–29.75). This disparity could be related to differences in study methodology, sample size, and study participants' sociocultural backgrounds. Likewise, poor documentation and record-keeping can result in a low prevalence of hepatotoxicity in a retrospective analysis. A subgroup analysis depending on the year of publication was also undertaken. According to this meta-analysis, the overall pooled prevalence of hepatotoxicity across studies completed between 2008 and 2014 G.C. was 27.59% (95% CI: 19.11–36.08). This is higher than the pooled prevalence of hepatotoxicity among the recently conducted studies, which is 22.29% (95% CI: 15.82–30.10). This decrease in hepatotoxicity over the last year could be attributed to the development of our health system, the growth of health facilities, the reduction of opportunistic infections, and the careful monitoring of HIV patients for organ toxicity and injuries.

In this meta-analysis, we attempted to conduct a subgroup analysis based on HIV/Tb co-infection status. According to a random effect meta-analysis, the total pooled prevalence of hepatotoxicity among HIV/Tb co-infected adults (26.3%) was higher than that of non-co-infected people (17.94%). Because the HIV virus weakens the immune system, HIV-positive people are more likely to develop active tuberculosis (Tb), which can exacerbate liver damage. Several prior studies [41, 75–79] have reported the hepatotoxicity of anti-Tb medications, which supports our finding. For example, a study in Cameroon discovered that 52.9% of Tb/HIV co-infected people develop hepatotoxicity [58]. On the other hand, a study in China found that 4.2% of HIV/Tb co-infected patients had hepatotoxicity [80]. This underscores the significance of closer and more frequent monitoring of liver function and other organs in individuals using anti-Tb drugs.

The presence of publication bias in this meta-analysis was revealed by the symmetry of the funnel plot and Egger’s test results. The sensitivity analysis, on the other hand, revealed that no single study had an impact on the total pooled effect size. Despite the fact that research selection criteria were well established and study selection was carried out by three independent reviewers, we were unable to completely eliminate publication bias, which may have influenced our findings. The following restrictions apply to this study: Even after subgroup analysis for multiple variables, there is still a lot of heterogeneity. Due to inconsistent risk factor assessments by the recruited studies, it was unable to examine parameters such as CD4 T cell count, sex, age, underlying illness status, type of HAART regimen, and HAART regimen duration that were linked to the pooled prevalence of hepatotoxicity in HIV-infected patients.

## Conclusion

According to the current systematic review and meta-analysis, hepatotoxicity is prevalent among HIV-infected people. People who had HIV/Tb co-infection and were on antiretroviral therapy (ART) had a higher overall pooled prevalence of hepatotoxicity. Many anti-HIV and anti-Tb medications have the potential to harm the liver. This comprehensive result emphasizes the significance of regular monitoring in order to avoid organ damage, particularly hepatotoxicity and HIV/AIDS-related complications. The findings of this study will also assist policymakers and other stakeholders. Furthermore, the data could be used in future research and decision-making based on evidence.

## Supplementary Information

**Additional file 1: **PRISMA 2020 checklist.

**Additional file 2: **Quality assessment form for included studies.

**Additional file 3: **The syntax for each database.

## Data Availability

All the datasets used and/or analyzed during the current study are available in the manuscript.
