# Supplementary material for: Prevalence of hepatotoxicity among HIV-infected patients in Ethiopia: a systematic review and meta-analysis
Source: BMC Infect Dis. 2022 Nov 9;22:826. doi: 10.1186/s12879-022-07838-w (PMC9647905; doi:10.1186/s12879-022-07838-w)
Supplement: Supplementary file 3 — Additional file 3: The syntax for each database. [file 12879_2022_7838_MOESM3_ESM.docx]

**PubMed**

(((Hepatotoxicity OR liver enzyme elevation OR "biochemical alteration" OR "antiretroviral therapy")) AND (("HIV/AIDS")) AND ((Ethiopia* OR Addis Ababa* OR Dire Dawa* OR east Ethiopia* OR Harari Region* OR Somali Region* OR northeast Ethiopia* OR north Ethiopia* OR Tigray Region* OR Afar Region* OR northwest Ethiopia* OR west Ethiopia* OR southwest Ethiopia* OR South West Ethiopia Peoples' Region* OR Southern Nations, Nationalities, and Peoples' Region* OR Sidama Region* OR Oromia Region* OR Gambela Region* OR Benishangul-Gumuz Region* OR Amhara Region* OR southeast Ethiopia* OR south Ethiopia*)) AND human NOT animal

**Google scholar**

“Hepatotoxicity OR liver enzyme elevation OR biochemical alteration AND HIV/AIDS’’ AND “Name each Ethiopian region”

**Other sources**

“Hepatotoxicity” and Ethiopia; “liver enzyme elevation” and Ethiopia; biochemical alteration and Ethiopia; “antiretroviral therapy” and hepatotoxicity and Ethiopia; “hepatotoxicity” and HIV/AIDS and Ethiopia; “liver enzyme elevation” and HIV/AIDS and Ethiopia; “biochemical alteration” and HIV and Ethiopia; ‘‘Hepatotoxicity’’ and “Ethiopian regions.”
